# Modern Sociology

**Published:** 1900-10-20

**Authors:** 


### Modern Sociology.

### MANCHESTER HEALTH MISSIONARIES.

About forty years ago some ladies in Manchester, realising the ignorance of women of the poorer classes regarding the elements of hygiene, and their indifference to personal and household cleanliness, resolved to give what instruction they could in these matters. They began by distributing health tracts, but soon found out that these were rarely read, and still more rarely acted upon. They then began to send out women who had received some training in hygiene, to give personal demonstrations. This was the beginning of the Manchester and Salford Sanitary Association. The Association now employs twenty-three paid women workers, named health visitors, in addition to a number of lady district superintendents and health lecturers, whose work is voluntary.

The health visitors are superior women of the class to be aided. Thus they comprehend, better than a woman of another class could do, the limitations of those whom they visit, and can speak to them more freely about their defects than—such is human nature—a person of higher social station could do without giving offence. Moreover, the home of the health visitor—a cottage or even a couple of rooms within the bounds of the district where she works—is a perpetual object lesson. She has no servants to do her work for her, yet her home is clean and comfortable; and this though she is out at work visiting in her district for six hours a day, and has daily reports to write for the medical officers of health, and weekly ones for her superintendent. If, with all this, she still finds time to keep her house spotless, there can be little excuse for other women not doing the same. The argument, "You don't know nothin' about it," which would meet a rich woman who tried to inculcate the duty of cleanliness to a working woman, cannot be flung at the health visitor, who is a working woman herself.

The duties of the health visitors are to go to every house, as the superintendents direct, irrespective of creed or circumstances. They carry with them carbolic powder, which they give to all who will accept it, and explain how it is to be used. This powder is provided, free of charge, by the sanitary authorities of Manchester and Salford, who appreciate the good work done by the Association. These authorities also provide brushes and lime for whitewashing purposes. The visitors give the lime to those who need it, but only lend the brushes, after giving instructions in their use. The esteem in which the Association is held is further shown by the fact that the Corporation of Manchester pays for nine, and that of Salford for four of the Association's visitors. The payment of the remainder is met by voluntary subscriptions.

The visitors direct the attention of those on whom they call to bad smells, tolerance of which by the inmates of the house has been bred by custom. They decide whether these are due to defective drainage or to lack of ventilation, and induce the house-mother to open her windows and to flush her drains, as well as to refrain from using the latter, instead of an ashpit, for the disposal of rags, potato skins, and refuse of all sorts. They advise as to the feeding and clothing of children, and where there is sickness they may make a bed and help to make the invalid comfortable. They make no pretensions to be regarded as nurses, and they are expected to report all cases of illness to the medical authorities, but they often find cases where a little help can be given at once. Therefore they carry with them some linseed meal, a pot of ointment, and a few bandages in their bags, and can make a poultice or bind up a wound if required.

Clubs of various kinds are formed in each district—coal clubs, blanket clubs, and general thrift clubs. These are much appreciated, as the women hardly miss the little sums they give every week, and when the accumulations come back in the form of coals or blankets, they feel as if they had received a gift. Every effort is made to induce them to attend the mothers' meetings which are held in each district. There the lady superintendents or some of the lecturers give a short talk on some subject connected with health, besides interesting themselves generally in the welfare of their protégées. Advice is always given them to attend faithfully at their respective places of worship, and to send their children regularly to school. On behalf of the medical authorities, the visitors distribute leaflets on such subjects as the Prevention of Consumption, Precautions against Summer Diarrhœa, Measles, or Whooping-cough, How Infants should be Fed, Suggestions to Householders, and the like. If merely handed in these leaflets would probably be left unread; but the visitor who gives them will talk the matter over with the recipient, and press home the information given in them.

During the twelve months ending November 1898, the last for which the statistics are yet published, the health visitors paid 35,966 visits in Manchester and 23,205 in Salford. Of the Manchester visits, 1,853 are reported as being made specially for the medical officers of health. An analysis of the visits paid shows that 6 per cent. of the houses visited were found to be dilapidated, 12 per cent. were dirty, 17 per cent. had improved since a recent visit, while 17 per cent. had been invaded by sickness, and 1 per cent. was overcrowded. An improvement of 17 per cent. of the houses of the poor in point of cleanliness means a great addition to the welfare of the occupants and a considerable improvement in the sanitary condition of the city at large. As one member of the Association, Mrs. Redford, says in speaking of its work: "It will readily be admitted that the best efforts of the most enlightened and progressive municipal authorities will be thwarted and hindered most seriously, unless the majority of the people are taught to make use of the many advantages they now possess, such as a plentiful supply of good water, baths, libraries, parks, &c.; and particularly taught also to keep their own dwellings and persons in a state of healthy cleanliness." This is the work which the Sanitary Association seeks to do—to go into the homes of the poor and ignorant, and instruct them in the first principles of healthy living. It might well be a subject for marvel that in spite of all the efforts of the municipal authorities in our large towns, preventible disease remains so constant. But though the authorities may provide water, they cannot compel people to use it; they may cleanse the streets, but they cannot, except in case of infectious disease, penetrate into the houses. This is what the Manchester Ladies' Sanitary Association is trying to do, and apparently with considerable success.

